# Die Anwendung antiallergisch beschichteter Knieendoprothesen ist mittelfristig sicher: Eine randomisierte kontrollierte Studie

**DOI:** 10.1007/s00132-021-04186-6

**Published:** 2021-11-03

**Authors:** Anne Postler, Franziska Beyer, Cornelia Lützner, Eric Tille, Jörg Lützner

**Affiliations:** UniversitätsCentrum für Orthopädie, Unfall- und Plastische Chirurgie, Universitätsklinikum Carl Gustav Carus Dresden, TU Dresden, Fetscherstraße 74, 01307 Dresden, Deutschland

**Keywords:** Allergie, Beschichtete Implantate, Metallunverträglichkeit, Metallionen, Knietotalendoprothese, Kniegelenkersatz, Allergy, Coated implant, Metal hypersensitivity, Metal ions, Total knee arthroplasty, Total knee replacement

## Abstract

**Hintergrund:**

Patienten mit einer Kontaktallergie gegen Implantatbestandteile erhalten in Deutschland üblicherweise beschichtete Prothesen. Ob die Versorgung mit solchen hypoallergenen Implantaten vergleichbare Ergebnisse erzielt wie die Standardversorgung mit Implantaten aus Kobalt-Chrom-Legierungen (CoCr) ist international umstritten und mittelfristig bislang wenig untersucht.

**Ziel der Arbeit (Fragestellung):**

Gibt es Unterschiede hinsichtlich Metallionenkonzentration im Blut, Kniefunktion und patientenberichteter Ergebnisse (PROM) zwischen beschichteten Knieendoprothesen und Standardimplantaten?

**Material und Methoden:**

118 Patienten erhielten randomisiert entweder eine beschichtete oder eine Standard-Knieendoprothese und wurden hinsichtlich Kniefunktion und PROM untersucht. Präoperativ, ein und 5 Jahre nach der Operation wurden zusätzlich die Metallionenkonzentrationen für Chrom, Kobalt, Molybdän und Nickel im Blut gemessen.

**Ergebnisse:**

Nach 5 Jahren konnten die Ergebnisse von 97 Patienten ausgewertet werden. Sowohl die Metallionenkonzentrationen als auch die Ergebnisse für PROM zeigten gleich gute Werte und keine Unterschiede zwischen den Gruppen. Während nach einem Jahr ein Anstieg der Chrom-Konzentration bei 13 Patienten über 2 µg/l im Plasma zu verzeichnen war, lag nach 5 Jahren kein gemessener Wert über 1 µg/l.

**Diskussion:**

Die Anwendung beschichteter Implantate gilt als umstritten und möglicherweise sogar als unsicher. In der vorliegenden Arbeit konnten mittelfristig gleich gute Ergebnisse zwischen beschichteten (TiNbN) und Standardprothesen (CoCr) nachgewiesen werden, sodass sich bei der Verwendung beschichteter Knieendoprothesen hinsichtlich gemessener Metallionenkonzentration und PROM kein Nachteil ergibt.

## Abstract (en)

**Background:**

In Germany, patients with contact allergy to implant components usually receive coated joint arthroplasties. Whether the treatment using these hypoallergenic implants achieves comparable results to standard treatment with implants consisting of cobalt-chromium alloy (CoCr) implants is controversially discussed internationally and has rarely been investigated in the mid-term.

**Objectives:**

Are there differences in blood metal ion concentrations, knee function, and patient-reported outcomes (PROM) between coated and standard implants?

**Material and methods:**

118 patients were randomized to receive either a coated or a standard implant. Knee function as well as patient-reported outcome measures were assessed. Metal ion concentrations in blood samples were additionally determined for chromium, cobalt, molybdenum, and nickel, preoperatively, one and five years after surgery.

**Results:**

After five years, it was possible to analyse the results of 97 patients. In metal ion concentrations, as well as PRO, consistently good results were seen, without any difference between the groups. While in 13 patients there was an increase in chromium concentration above 2 µg/l one year after surgery, there was no measured value above 1 µg/l after five years.

**Conclusion:**

In our study, similar mid-term results were detected for coated (TiNiN) and standard (CoCr)TKA. With respect to metal ion concentrations and PRO there are no disadvantages in using coated TKA.

## Kurze Hinführung zum Thema

Leiden Patienten an einer Kontaktallergie gegen Implantatbestandteile, fragen sie im Vorfeld ihrer Operation oft nach den verwendeten Materialen und werden in Deutschland häufig mit beschichteten Implantaten versorgt [1]. Ob diese beschichteten Knieendoprothesen vergleichbare Ergebnisse erreichen wie Standardprothesen, ist wenig untersucht. Im Rahmen einer randomisiert kontrollierten Studie sollten daher die Metallionenkonzentration sowie die patientenberichteten Ergebnisse nach Implantation von beschichteten und Standardknietotalendoprothesen verglichen werden.

## Hintergrund und Fragestellung

Die Wahl des Implantates ist elementarer Bestandteil der Operationsplanung einer Knieendoprothese. Neben Kopplungsgrad und Design spielen auch die Materialien eine wichtige Rolle. Üblicherweise werden Implantate aus Kobalt-Chrom-Legierungen verwendet. Nach deren Implantation kommt es immer zu Freisetzung einzelner Materialbestandteile. Hierbei spielen v. a. elektrochemisch (Korrosion) bedingte Freisetzungen sowie deren Überlagerung in der feuchten Körperumgebung eine Rolle. Mechanisch bedingte Freisetzungen (Verschleiß) sind seltener. Grundsätzlich führt die Freisetzung der Ionen der enthaltenen Metalle zu einer biologischen Reaktion beim Patienten [18]. Das Ausmaß der Ionenkonzentration ist hierbei von der Höhe der Korrosionsanfälligkeit des jeweiligen Metalls im Körpermilieu abhängig [3, 32].

In der Hüftendoprothetik gewann dieses Problem bei der Verwendung von Metall-Metall-Gleitpaarungen an Bedeutung, als infolge von Metallabrieb ausgeprägte lokale und systemische toxische Effekte beobachtet wurden. Diese Gleitpaarung wird deswegen nicht mehr empfohlen [13]. Es besteht daher die Notwendigkeit, sich mit den Implantatmaterialien auseinanderzusetzen, insbesondere wenn Patienten angeben, an einer Kontaktallergie gegen Metalle zu leiden. Eine implantatbezogene Unverträglichkeit gilt als Typ-IV-Reaktion mit verzögerter zellvermittelter Immunreaktion. Dabei bleibt nach wie vor unklar, ob die Allergietestung mittels Epikutantest eine tatsächliche Überempfindlichkeit im Gelenk vorhersagen kann oder ob überhaupt ein Zusammenhang besteht [12].

Die Prävalenz von Kontaktallergien gegen Nickel wird mit bis zu 20,4 %, für Kobalt mit 3,4 % und für Chrom mit 1,5 % angegeben [19]. Gegen die übrigen Implantatbestandteile sind Allergien sehr selten. Patienten mit Kontaktallergien fragen häufig im Vorfeld ihrer Operation nach „Allergieimplantaten“. Meist werden in der Knieendoprothetik bei Patienten mit einer entsprechenden Hautallergie hypoallergene Implantate verwendet. Es ist auch möglich und international üblich, trotz bekannter Allergie Standardprothesen zu verwenden, wie auch vom AK 20 der Deutschen Gesellschaft für Orthopädie und Orthopädische Chirurgie (DGOOC), der Deutschen Kontaktallergie-Gruppe (DKG) und der Deutschen Gesellschaft für Allergologie und Klinische Immunologie (DGAKI) publiziert [30]. Dabei sind jedoch in Deutschland die Patienten zwingend auf die Möglichkeit der Verwendung von Alternativmaterialien hinzuweisen und deren Zustimmung zur Verwendung einer Standardprothese zu dokumentieren [39]. Diese Zustimmung wird bei vielen Patienten nicht erzielt werden können, da Allergiepatienten häufig unter Angststörungen leiden [6, 23]. Insofern ist für diese Patienten ein hypoallergenes Implantat notwendig.

In der Hüftendoprothetik kann auf den Einsatz von Kobalt-Chrom-Legierungen zugunsten von Titan-Legierungen weitgehend verzichtet werden. In der Knieendoprothetik besteht zumindest die Femurkomponente fast immer aus einer Kobalt-Chrom-Molybdän-Legierung mit sehr geringen Anteilen von Nickel (ISO 5832-4). Soll der Metallkontakt vermieden werden, muss die Oberfläche beschichtet oder keramisiert oder ein System mit keramischer Femurkomponente verwendet werden. Am häufigsten werden in Deutschland beschichtete oder keramisierte Implantate eingesetzt [5]. Bei der Beschichtung gibt es dabei Einfachbeschichtungen und Mehrfachbeschichtungen, die sich durch eine besonders hohe mechanische Stabilität auszeichnen [26]. Die Beschichtung soll dabei die Metallionenfreisetzung und eine sich daraus bei entsprechender Disposition ergebende mögliche Gefahr von Unverträglichkeiten reduzieren. Ob diese teureren beschichteten Endoprothesen vergleichbare Ergebnisse zu den etablierten Standardendoprothesen erzielen und somit bei entsprechender Notwendigkeit eines hypoallergenen Implantates unbedenklich eingesetzt werden können, ist international umstritten und im längeren Follow-up bislang kaum untersucht [12].

Aus diesem Grund wurde die Studie initiiert, um Metallionenkonzentration im Blut, Kniefunktion und patientenberichtete Ergebnisse (PROM) für beschichtete Knieendoprothesen im Vergleich mit Standardimplantaten prospektiv zu untersuchen.

## Studiendesign und Untersuchungsmethoden

Alle Patienten, die zwischen 2012 und 2015 eine Knieendoprothese (Knie-TEP) erhalten haben, wurden hinsichtlich eines Einschlusses in die Studie geprüft. Ausschlusskriterien waren eine bekannte Kontaktallergie gegen Metalle, ein vorhandenes Metallimplantat oder die Notwendigkeit eines Implantates mit höherem Kopplungsgrad. Nach schriftlicher Einwilligung zur Teilnahme, erfolgte die randomisierte Zuordnung von 122 Patienten für eine ungekoppelte beschichtete oder Standard-Knie-TEP. Das Implantatdesign war bis auf die Beschichtung identisch.

Erfasst wurden dabei der Oxford Knee Score (OKS) [4, 21], der Short Form 36 (SF-36) [9] und die University of Los Angeles Activity Rating Scale (UCLA) [38] sowie Komplikationen.

**Figure 1:**
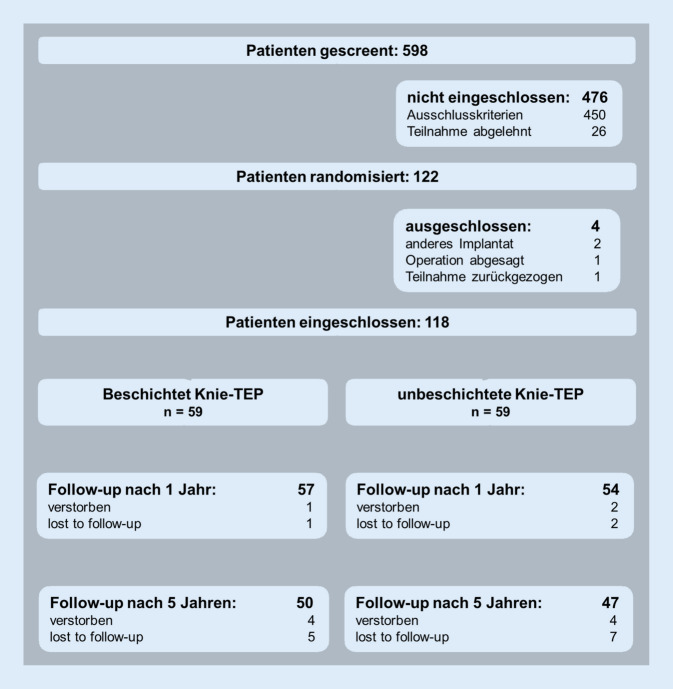

Insgesamt wurden 111 Patienten zum 1‑Jahres-Follow-up, einschließlich der Metallionenanalyse, nachuntersucht. Die PROM wurden postalisch zum 3‑Jahres-Follow-up von 103 Patienten erhoben und nach 5 Jahren erfolgte die Nachuntersuchung von 97 Patienten mit Follow-up der Metallionenanalyse (Abb. 1).

Im mittelfristigen Verlauf sind insgesamt acht Patienten – ohne direkten Zusammenhang zur Operation der Knieendoprothese – gestorben. Vier Patienten waren aufgrund anderer Krankheiten nicht mehr in der Lage, die Fragebögen zu beantworten und acht Patienten haben die weitere Studienteilnahme abgelehnt („lost to follow-up“ von 12 Patienten).

Für alle Patienten wurde die balanSys-Knieendoprothese (Mathys, Bettlach, Schweiz) verwendet. Diese Knieendoprothesen bestehen aus einer Kobalt-Chrom-Legierung gemäß der Norm ISO 5832‑4 (26,5–30,0 % Chrom, 4,5–7,0 % Molybdän, bis zu 1,0 % Nickel, bis zu 1,0 % Eisen, bis zu 0,35 % Carbon, bis zu 1,0 % Mangan, bis zu 1,0 % Silikon und der verbleibende Anteil Kobalt). Die Einfachbeschichtung, die dem gesamten Implantat durch physikalische Gasphasenabscheidung („physical vapour deposition“) mit einer Dicke von 4,5 µg aufgetragen ist, besteht aus Titan-Niob-Nitrid und hat eine hohe Abriebfestigkeit mit niedrigem Reibungskoeffizienten. Für alle Patienten wurde ein ultrahochmolekulargewichtiges Polyethylen(UHMWPE)-Inlay verwendet. Nach einer Single-Shot-Gabe eines Antibiotikums (1,5 g Cefuroxim) wurden alle Operationen in pneumatischer Blutsperre, unter Anwendung eines medialen parapatellaren Zugangs und einer konventionell gemessenen Resektionstechnik durchgeführt. Alle Endoprothesen wurden zementiert und immer ein „Fixed-bearing“-Inlay verwendet. Ein Retropatellarersatz hingegen wurde nicht implantiert. Ab dem 1. postoperativen Tag wurden die Patienten unter Vollbelastung mobilisiert.

Die Serumproben für die Metallionenanalyse wurden präoperativ sowie zum 1‑ und 5‑Jahres-Follow-up in 7,5 ml S‑Monovetten® entnommen (Sarstedt AG, Nümbrecht, Deutschland). Dabei wurde eine spezielle Kanüle für die Metall-Spurenanalytik verwendet (Sarstedt AG). Innerhalb der ersten Stunde nach der Probenentnahme wurde das Plasma durch 10-minütiges Zentrifugieren bei 2500 g getrennt. Die Proben wurden bei −20 °C aufbewahrt, bevor sie mittels Graphitrohr-Atomabsorptionsspektrometrie (Z-8270 mit Polarisation-Zeeman-Absorption; Hitachi Ltd., Tokio, Japan) auf den Chrom‑, Kobalt‑, Molybdän- und Nickel-Gehalt mit einem analysiert wurden.

Die Genauigkeit der Methode wurde validiert mit einer < 10 %igen Variabilität, anhand von Kontrollproben SeronormTM Trace Elements Serum (SERO AS, Billingstad, Norwegen). Die Nachweisgrenze des Verfahrens wurde auf 0,5 µg/l für Chrom, Kobalt und Molybdän sowie 1,0 µg/l für Nickel geschätzt (Mittelwert +3 Standardabweichungen des Puffers). Alle Proben, die Ionenwerte unter der Nachweisgrenze aufwiesen, wurden für Chrom, Kobalt und Molybdän auf 0,25 µg/l angeglichen und auf 0,5 µg/l für Nickel. Die Studie wurde von der Ethikkommission der TU Dresden befürwortet (EK 339092011).

Alle Daten der Fragebögen der Patientenakten wurden in eine elektronische Datenbank eingepflegt und in SPSS (27 für Windows) zur Analyse übertragen. Die deskriptive Auswertung basiert auf Mittelwerten und Standardabweichungen für kontinuierliche Variablen und absoluten sowie relativen Häufigkeiten für kategorische Variablen. Für die Werte, die keiner Normalverteilung folgen (Metallionenkonzentration), wurde der Median verwendet. Der Vergleich zwischen den Gruppen wurde mittels des t‑Test für kontinuierliche Variablen und mittels Chi-Quadrat-Test für kategorische Variablen durchgeführt. Die Metallionenkonzentration folgten keiner Normalverteilung, da viele Werte unter der Nachweisgrenze lagen, sodass die Gruppen unter Anwendung des Mann-Whitney-U-Tests verglichen wurden. Für den Vergleich der Werte unter oder über dem Cut-off von 2 µg/l wurde der Chi-Quadrat-Test verwendet. Das Signifikanzniveau wurde auf *p* < 0,05 festgelegt. Der primäre Endpunkt war die Metallionenkonzentration und der sekundäre Endpunkt die PROM sowie die unerwünschten Ereignisse. Um Unterschiede der Metallionenkonzentration von 1 µg/l bei einem Signifikanzniveau von 5 % und einer Power von 80 % nachzuweisen, wurde ein Minimum von 36 Patienten pro Gruppe kalkuliert. Um auch einen möglichen „lost to follow-up“ in dieser älteren Patientengruppe zu erlauben, wurde eine Gesamtzahl von 60 Patienten pro Gruppe angestrebt.

## Ergebnisse

Die Gruppen der beschichteten und der Standardknieendoprothesen waren bzgl. präoperativer und postoperativer Daten hinsichtlich Geschlecht, Alter, BMI, stationärer Verweildauer, Schnitt-Naht-Zeiten und Komorbiditäten vergleichbar (siehe Tab. 1 für Details).

**Table 1:** 

		Beschichtet (*n* = 50)	Standard (*n* = 47)	*p*-Wert
Geschlecht	Männlich	20 (40,0 %)	19 (40,4 %)	0,966
	Weiblich	30 (60,0 %)	28 (59,6 %)	
ASA-Klassifikation	1 + 2	36 (72,0 %)	27 (57,4 %)	0,133
	3 + 4	14 (28,0 %)	20 (42,6 %)	
Alter (Jahre)		66,6 (9,6)	67,2 (9,5)	0,742
BMI (kg/m^2^)		31,5 (4,7)	32,2 (5,6)	0,497
Schnitt-Naht-Zeit (min)		78,5 (10,7)	78,8 (12,9)	0,891

*ASA* American Society of Anesthesiologists

### Unerwünschte Ereignisse

Ein Patient erlitt 13 Tage nach Primärimplantation einer beschichteten Knie-TEP eine traumatische Wunddehiszenz, die zur Revision mit Inlaywechsel führte. Sieben Monate nach Primärimplantation einer Standard-Knie-TEP musste bei einem weiteren Patienten die Explantation infolge eines periprothetischen Infektes durchgeführt werden. Nach 1,8 Jahren erfolgte bei einem Patienten mit beschichteter Knie-TEP die Arthroskopie mit Resektion einer Plica synovialis.

### Metallionenkonzentrationen

Bei der Chrom-Konzentration im Patientenplasma gab es einen Anstieg im ersten postoperativen Jahr von im Median 0,25 auf 1,30 µg/l in der Standardgruppe, geringer ausgeprägt auch bei den beschichteten Implantaten. Nach 5 Jahren war dieser Anstieg wieder vollständig rückläufig auf einen medianen Wert von 0,25 µg/l, sowohl in der Gruppe der Standard- als auch der beschichteten Knieendoprothesen.

**Figure 2:**
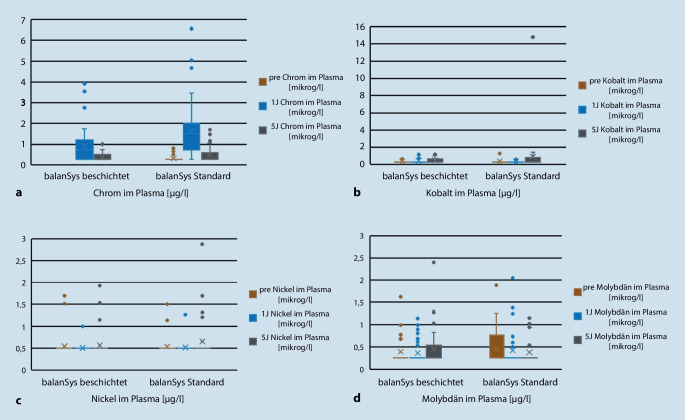

Die Konzentrationen von Kobalt, Molybdän und Nickel änderten sich in beiden Gruppen nicht (Tab. 2; Abb. 2).

**Table 2:** 

		Beschichtet	Standard	*p*-Wert
Chrom	Präoperativ	0,25 (0,25; 0,25)	0,25 (0,25; 0,25)	n. s.
	1 Jahr postoperativ	0,74 (0,25; 1,21)	1,30 (0,63; 1,82)	*p* = 0,005
	5 Jahre postoperativ	0,25 (0,25; 0,50)	0,25 (0,25; 0,60)	n. s.
Kobalt	Präoperativ	0,25 (0,25; 0,25)	0,25 (0,25; 0,25)	n. s.
	1 Jahre postoperativ	0,25 (0,25; 0,25)	0,25 (0,25; 0,25)	n. s.
	5 Jahre postoperativ	0,25 (0,25; 0,63)	0,25 (0,25; 0,69)	n. s.
Molybdän	Präoperativ	0,25 (0,25; 0,25)	0,25 (0,25; 0,68)	n. s.
	1 Jahre postoperativ	0,25 (0,25; 0,25)	0,25 (0,25; 0,25)	n. s.
	5 Jahre postoperativ	0,25 (0,25; 0,54)	0,25 (0,25; 0,25)	n. s.
Nickel	Präoperativ	0,50 (0,50; 0,50)	0,50 (0,50; 0,50)	n. s.
	1 Jahre postoperativ	0,50 (0,50; 0,50)	0,50 (0,50; 0,50)	n. s.
	5 Jahre postoperativ	0,50 (0,50; 0,50)	0,50 (0,50; 0,50)	n. s.

Ein Jahr nach der Implantation ihrer Knieendoprothese zeigten sich bei 13 Patienten (drei mit beschichteter, zehn mit Standardprothese, *p* = 0,040) Plasmawerte für Chrom über 2 µg/l, was als möglich kritische Grenze für Metall-Metall-Gleitpaarungen („metal-on-metal“ [MoM]) in der Hüftendoprothetik betrachtet wird [13]. Einer dieser Patienten konnte zum 5‑Jahres-Follow-up nicht erreicht werden. Die weiteren zwölf Patienten zeigten vollständig rückläufige Werte auf unter 1 µg/l nach 5 Jahren.

### Patientenberichtete Ergebnisse (PROM)

In allen Scores zeigte sich über 5 Jahre postoperativ eine substanzielle Verbesserung mit der höchsten Änderung im ersten postoperativen Jahr. Zwischen den Gruppen der Standard- und der beschichteten Knieendoprothesen bestanden keine Unterschiede (Tab. 3).

**Table 3:** 

		Beschichtet	Standard	*p*-Wert
Oxford-Score (0–48 Punkte)	Präoperativ	21,3 (6,0)	20,6 (6,3)	n. s.
	1 Jahr postoperativ	38,7 (6,8)	37,6 (7,4)	n. s.
	5 Jahre postoperativ	40,0 (6,9)	39,4 (5,6)	n. s.
OKS Pain Component Subscale (0–100 Punkte)	Präoperativ	40,0 (13,8)	40,2 (14,2)	n. s.
	1 Jahr postoperativ	84,5 (16,4)	81,6 (16,8)	n. s.
	5 Jahre postoperativ	87,3 (16,0)	86,8 (13,4)	n. s.
OKS Function Component Subscale (0–100 Punkte)	Präoperativ	50,6 (13,7)	46,8 (14,6)	n. s.
	1 Jahr postoperativ	75,3 (14,4)	73,6 (15,2)	n. s.
	5 Jahre postoperativ	77,6 (14,6)	75,0 (13,6)	n. s.
UCLA (Level 1–10)	Präoperativ	3,9 (1,4)	3,8 (1,6)	n. s.
	1 Jahr postoperativ	4,9 (1,5)	5,0 (1,4)	n. s.
	5 Jahre postoperativ	5,1 (1,4)	4,9 (1,6)	n. s.
SF-36 Körperliche Summenskala	Präoperativ	26,3 (7,7)	25,3 (6,4)	n. s.
	1 Jahr postoperativ	43,6 (12,1)	43,1 (10,1)	n. s.
	5 Jahre postoperativ	43,9 (9,7)	41,5 (12)	n. s.
SF-36 Psychische Summenskala	Präoperativ	49,6 (13,4)	53,2 (14,7)	n. s.
	1 Jahr postoperativ	53,0 (10,5)	51,4 (12,9)	n. s.
	5 Jahre postoperativ	51,7 (12,3)	51,6 (11,7)	n. s.

*OKS* Oxford Knee Score, *SF-36 *Short Form 36, *UCLA* University of Los Angeles Activity Rating Scale

**Figure 3:**
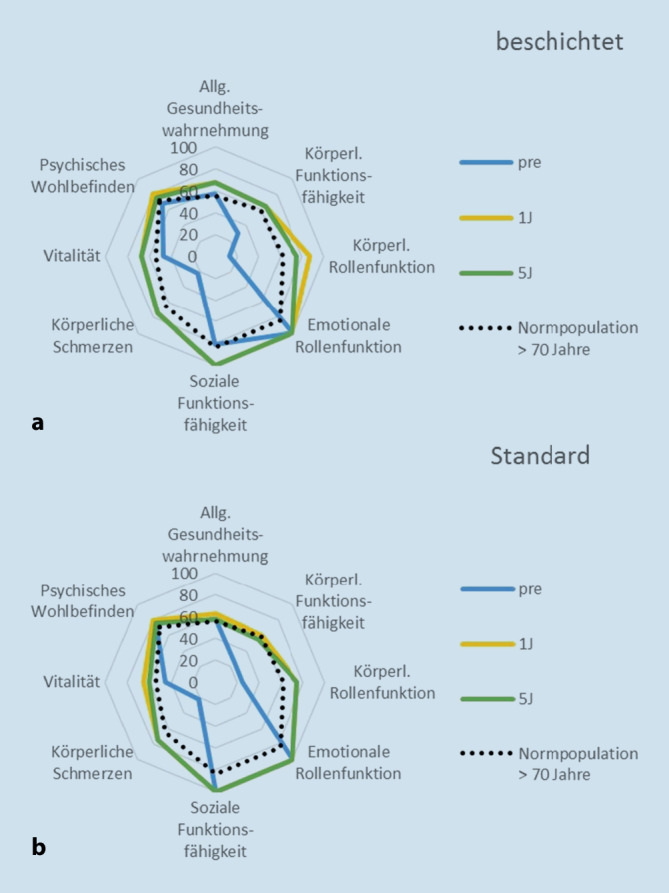

Die Ergebnisse der gesundheitsbezogenen Lebensqualität („health-related quality of life“) waren zum 1‑Jahres-Follow-up bereits ähnlich derer einer altersangepassten Normalpopulation (Abb. 3). Sowohl die körperliche als auch die psychische Summenskala zeigten einen deutlichen Anstieg innerhalb des ersten postoperativen Jahres, allerdings keine wesentliche Änderung bzw. nur einen leichten Rückgang vom 1‑Jahres- zum 5‑Jahres-Follow-up.

## Diskussion

Sowohl nach Implantation beschichteter als auch von Standardprothesen zeigten sich gute Ergebnisse im mittelfristigen Follow-up.

### Metallionenkonzentration

Während ein Jahr postoperativ bei der Messung der Metallionenkonzentration noch ein Anstieg der Chrom-Konzentration beobachtet wurde, und dieser ausgeprägter in der Standardgruppe (Patienten, die mit einer Standardendoprothese versorgt wurden), war dieser nach 5 Jahren vollständig rückläufig. Innerhalb des ersten Jahres wurde nur bei wenigen Patienten ein Anstieg auf über 2 μg/l gesehen (Grenzwert für MoM-Gleitpaarungen in der Hüftendoprothetik [31]).

Das Überschreiten dieses Grenzwertes wurde ein Jahr postoperativ häufiger in der Standardgruppe beobachtet (18 % vs. 5 %) und entspricht den Erwartungen bei der Verwendung beschichteter Implantatkomponenten. Nach 5 Jahren zeigten jedoch alle Patienten unabhängig von der Beschichtung wieder Werte für Chrom von unter 2 µg/l. Für Kobalt, welches die Hauptkomponente der Legierung ist, wurde hingegen nie ein Anstieg auf über 2 μg/l gesehen. Auch hier wurde in MoM-Studien von erhöhten Werten berichtet [15, 33, 36] und andere Arbeiten, die die Metallionenfreisetzung bei der Verwendung von Knieimplantaten untersucht haben, berichten über erhöhte Werte für Chrom und Kobalt bis zu 10 Jahre nach Implantation [7, 17, 27]. Für Nickel, das Metall, welches am häufigsten Hautallergien hervorruft (bis zu 20,1 % [28]) und daher in den Patientengesprächen oft benannt wird, wurden keine Änderungen der Konzentration bis zu 5 Jahre nach Operation in beiden Patientengruppen gemessen. Jedoch besteht auch nur weniger als 1,0 % des Implantates aus Nickel. Bestätigt wird das Ergebnis dieser unveränderten Konzentrationen durch Arbeiten, in denen Patienten mit einem positiven Epikutantest in der Allergietestung für Nickel mit einem Standardimplantat versorgt wurden, und dabei keine schlechteren postoperativen Ergebnisse zeigten [2, 19]. Insofern scheint die Bedeutung des Nickel-Anteils bei Standardlegierungen in der Knieendoprothetik gering zu sein.

### Einsatz hypoallergener Implantate

In einem englischen Konsensuspaper wurde mit mind. 60 %iger Übereinstimmung die Empfehlung ausgesprochen, Patienten vor einer endoprothetischen Versorgung nicht regelhaft nach einer Metallallergie zu befragen und Standardprothesen sowohl für angegebene Metallallergien mit negativem Epikutantest aber auch positivem Epikutantest für Kobalt, Chrom oder Nickel zu verwenden. Die Autoren weisen allerdings auf die fehlende Evidenz und die für diese Arbeit zugrunde liegenden Expertenmeinungen hin [25]. In Deutschland ist die Situation etwas anders. Hier sind Patienten vor einer elektiven Endoprothesenimplantation nach Allergien zu befragen und im Falle einer Allergie auf die Möglichkeit der Verwendung von Alternativmaterialien hinzuweisen [39].

Neben beschichteten Implantaten, wie in dieser Arbeit untersucht, kommen als hypoallergene Implantate auch oberflächengehärtete Werkstoffe (Härtung des Grundmaterials durch eine Sauerstoffdiffusionshärtung, bekannt unter dem Handelsnamen OXINIUM^TM^, Smith & Nephew, London, UK) und vollkeramische Komponenten zum Einsatz. Aus dem australischen Register ist allerdings bekannt, dass diese oberflächengehärteten Endoprothesen höhere Revisionsraten aufweisen, insbesondere höhere Lockerungsraten bei ≥ 75-Jährigen [37]. Ähnliche Ergebnisse fanden sich auch im EPRD. Auch hier konnte kein Vorteil von beschichteten Implantaten nachgewiesen werden [10]. Die Ursachen für diese höheren Revisionsraten trotz theoretisch besserer Abriebeigenschaften sind unklar. In der Literatur finden sich jedoch Hinweise, dass Patienten mit und ohne Allergie gegen Implantatmaterialien nicht die gleichen Voraussetzungen aufweisen und somit Allergiepatienten von vornherein ein höheres Revisionsrisiko haben [6, 10, 22, 23]. Insofern kann die Sicherheit einer Implantatbeschichtung nur an vergleichbaren Patienten beurteilt werden, was in dieser Arbeit erfolgt ist. Hier zeigten sich vergleichbare Ergebnisse.

### Patientenberichtete Ergebnisse (PROM)

Es existieren wenige andere Arbeiten, in denen Patienten mit beschichteten und mit Standardimplantaten verglichen wurden. Eine Arbeit zeigt gleich gute Ergebnisse für Schmerz und Funktion (KSS und OKS) in beiden Gruppen mit zementfreien Knieendoprothesen in einer 10-Jahres-Nachuntersuchung [16]. In unserer Untersuchung zeigen beide Patientengruppen im mittelfristigen Follow-up nach 5 Jahren ebenfalls gleichermaßen eine signifikante funktionelle Verbesserung. In beiden von uns untersuchten Gruppen wurde eine signifikante Verbesserung der gesundheitsbezogenen Lebensqualität nachgewiesen, die mit der einer altersangepassten Normpopulation vergleichbar ist [11]. Zwischen den beiden Gruppen in unserer Untersuchung konnten zu keinem Zeitpunkt signifikante Unterschiede in den erfassten Scores zu Funktion, Lebensqualität, Aktivität, aber auch im Auftreten von unerwünschten Ereignissen festgestellt werden.

Aufgrund der nicht eindeutigen Evidenzlage zur Verwendung von hypoallergenen Knieendoprothesen kommen einige Autoren zu der Einschätzung, dass deren Einsatz nicht gerechtfertigt ist [19, 29, 34]. Dies wird einerseits mit dem unsicheren Zusammenhang zwischen Kontaktallergie und Gelenkreaktion begründet, andererseits wird dabei aber auch die Sorge vor möglichen negativen Effekten wie Abplatzen der Beschichtung deutlich [8, 24, 35]. Solche negativen Effekte konnten in der vorliegenden Studie mit mittelfristigem Follow-up nicht beobachtet werden. In Deutschland ist es zwar grundsätzlich möglich, einen Patienten mit nachgewiesener Kontaktallergie gegen einen Implantatbestandteil mit einer Standardendoprothese zu versorgen, dies muss jedoch mit dem Patienten ausführlich besprochen und dokumentiert werden. In einer dänischen Auswertung aus Registerdaten wurde deutlich, dass bei Vorliegen eines positiven Epikutantests hinsichtlich einer Metallallergie bei der Implantation von Knieendoprothesen keine höheren Komplikations- oder Revisionsraten bestehen. Allerdings wiesen die Fälle mit mehrfachen Revisionen häufiger Allergien gegen Kobalt und Chrom auf. Die Arbeit berücksichtigt jedoch nicht die Art und Beschaffenheit der Endoprothesen [20].

Viele Patienten wünschen dann trotz Aufklärung über den unsicheren Nutzen die Verwendung einer „Allergieprothese“. Eine positive Anamnese hinsichtlich einer Metallallergie ging in einer Arbeit von Nam D et al. mit geringeren Werten im Knee Society Function Score, geringerer Erwartung und geringerer Zufriedenheit einher. Außerdem bestehen bei Allergiepatienten häufig psychologische Auffälligkeiten [6, 23]. Deren Zweifel sollten Chirurgen begegnen [22]. Auch in Deutschland kommen demnach viele Kliniken dem Wunsch einer „Allergieprothese“ nach, auch wenn die Zusatzkosten im DRG-System nicht vergütet werden. Unabhängig davon, ob man die Verwendung von hypoallergenen Knieendoprothesen bei Patienten mit einer entsprechenden Kontaktallergie für sinnvoll erachtet oder nicht, erfolgt dies in einem relevanten Anteil. In Deutschland wurden 2019 bei 8,6 % der primären Knieendoprothesen beschichtete oder keramisierte Implantate verwendet [5]. Dies scheint nach den Ergebnissen der vorliegenden Studie zwar kein Vorteil, aber auch kein Nachteil zu sein, wenn man den ökonomischen Aspekt der Mehrkosten unberücksichtigt lässt.

### Limitationen

Die Studie weist einige Limitationen auf. Aufgrund der Ausschlusskriterien sind keine Patienten mit einer bekannten Metallunverträglichkeit eingeschlossen worden, obwohl diese Patienten die eigentliche Zielgruppe für die Anwendung beschichteter Implantate darstellen und der Einfluss der passageren Erhöhung der Metallionenkonzentrationen bei diesen Patienten gemessen werden sollte. Dies war jedoch aufgrund der geltenden Empfehlungen in Deutschland nicht möglich. Andere Aspekte, wie mechanische Probleme oder mögliche aseptische Lockerungen aufgrund der Beschichtung, aber auch Unterschiede in PROM können auch an Patienten ohne Metallunverträglichkeit erfasst werden.

Die Messung der Metallionenkonzentration erfolgte im Patientenplasma. Ein Rückschluss auf die lokale Reaktion im Gelenk ist damit nicht sicher möglich. Aus Studien zu den MoM-Gleitpaarungen in der Hüftendoprothetik ist bekannt, dass kein ausreichender Zusammenhang zwischen den Blutwerten und einer lokalen adversen Gewebsreaktion („adverse reactions to metal debris“) besteht [14]. Ähnliche Studien in der Knieendoprothetik existieren nicht.

## Fazit für die Praxis

- Beschichtete und Standardknieendoprothesen zeigen mittelfristig vergleichbare Ergebnisse mit niedrigen Metallionenwerten im Patientenplasma.
- Auch wenn sich kein evidenzbasierter Vorteil durch die Beschichtung ergibt, kann man bei bekannter Kontaktallergie und auf Wunsch des Patienten die Implantation einer beschichteten Prothese anbieten.
- Nach aktuellem Kenntnisstand ergibt sich daraus kein Nachteil, aber auch kein Vorteil, von psychologischen Aspekten bei Allergiepatienten abgesehen.

## Abkürzungen

ASA: American Society of Anesthesiologists
BMI: Body-Mass-Index
CONSORT: Consolidated Standards of Reporting Trials
DGAKI: Deutsche Gesellschaft für Allergologie und Klinische Immunologie
DGOOC: Deutsche Gesellschaft für Orthopädie und Orthopädische Chirurgie
DKG: Deutsche Kontaktallergie-Gruppe
DRG: Diagnosis Related Groups
EPRD: Endoprothesenregister Deutschland
ISO: Internationale Organisation für Normung
KSS: Knee Society Score
MoM: „Metal-on-metal“
OKS: Oxford Knee Score
PROM: Patient-reported Outcome Measure (Patienten-berichtete Ergebnisse)
SF-36: Short Form 36
TEP: Totalendoprothese
UCLA: University of Los Angeles Activity Rating Scale
UHMWPE: Ultrahochmolekulargewichtiges Polyethylen

## References

[1] Bindawas SM (2016). Relationship between frequent knee pain, obesity, and gait speed in older adults: data from the osteoarthritis Initiative. Clin Interv Aging.

[2] Bravo D, Wagner ER, Larson DR (2016). No increased risk of knee arthroplasty failure in patients with positive skin patch testing for metal hypersensitivity: a matched cohort study. J Arthroplasty.

[3] Clarke E, Hickman J (1953). An investigation into the correlation between the electrical potentials of metals and their behaviour in biological fluids. J Bone Joint Surg Br.

[4] Dawson J, Fitzpatrick R, Murray D (1998). Questionnaire on the perceptions of patients about total knee replacement. J Bone Joint Surg Br.

[5] Endoprothesenregister Deutschland (2020). Jahresbericht 2020 Mit Sicherheit mehr Qualität.

[6] Ferrer T, Hinarejos P, Goicoechea N (2020). Anxiety is the cause of the worse outcomes of allergic patients after total knee arthroplasty. Knee Surg Sports Traumatol Arthrosc.

[7] Friesenbichler J, Maurer-Ertl W, Sadoghi P (2012). Serum metal ion levels after rotating-hinge knee arthroplasty: comparison between a standard device and a megaprosthesis. International Orthopaedics (SICOT).

[8] Galetz MC, Fleischmann EW, Konrad CH (2010). Abrasion resistance of oxidized zirconium in comparison with CoCrMo and titanium nitride coatings for artificial knee joints. J. Biomed. Mater. Res. Part. B. Appl. Biomater..

[9] Garratt A, Schmidt L, Mackintosh A (2002). Quality of life measurement: bibliographic study of patient assessed health outcome measures. BMJ.

[10] Grimberg AW, Grupp TM, Elliott J (2021). Ceramic coating in cemented primary total knee arthroplasty is not associated with decreased risk of revision due to early prosthetic joint infection. J Arthroplasty.

[11] Gunzelmann T, Albani C, Beutel M (2006). Subjective health of older people in view of the SF-36: values from a large community-based sample. Z Gerontol Geriatr.

[12] Hallab NJ, Jacobs JJ (2009). Biologic effects of implant debris. Bull NYU Hosp Jt Dis.

[13] Hannemann F, Hartmann A, Schmitt J (2013). European multidisciplinary consensus statement on the use and monitoring of metal-on-metal bearings for total hip replacement and hip resurfacing. Orthop Traumatol Surg Res.

[14] Hartmann A, Kieback J-D, Lützner J (2017). Adverse reaction to metal debris in a consecutive series of DUROM™ hip resurfacing: pseudotumour incidence and metal ion concentration. Hip Int.

[15] Lainiala OS, Moilanen TP, Hart AJ (2016). Higher blood cobalt and chromium levels in patients with unilateral metal-on-metal total hip arthroplasties compared to hip resurfacings. J Arthroplasty.

[16] Louwerens JK, Hockers N, Achten G (2021). No clinical difference between TiN-coated versus uncoated cementless CoCrMo mobile-bearing total knee arthroplasty; 10-year follow-up of a randomized controlled trial. Knee Surg Sports Traumatol Arthrosc.

[17] Luetzner J, Krummenauer F, Lengel AM (2007). Serum metal ion exposure after total knee arthroplasty. Clin Orthop Relat Res.

[18] Lützner J, Günther K-P, Postler A (2020). Metal Ion release after hip and knee arthroplasty—causes, biological effects and diagnostics. Z Orthop Unfall.

[19] Middleton S, Toms A (2016). Allergy in total knee arthroplasty: a review of the facts. Bone Joint J.

[20] Münch HJ, Jacobsen SS, Olesen JT (2015). The association between metal allergy, total knee arthroplasty, and revision: study based on the Danish knee arthroplasty register. Acta Orthop.

[21] Naal FD, Impellizzeri FM, Sieverding M (2009). The 12-item oxford knee score: cross-cultural adaptation into German and assessment of its psychometric properties in patients with osteoarthritis of the knee. Osteoarthritis Cartilage.

[22] Nam D, Li K, Riegler V (2016). Patient-reported metal allergy: a risk factor for poor outcomes after total joint arthroplasty?. J Arthroplasty.

[23] Peña P, Ortega MA, Buján J (2021). Influence of psychological distress in patients with hypoallergenic total knee arthroplasty. Treatment algorithm for patients with metal allergy and knee osteoarthritis. Int J Environ Res Public Health.

[24] Raimondi MT, Pietrabissa R (2000). The in-vivo wear performance of prosthetic femoral heads with titanium nitride coating. Biomaterials.

[25] Razak A, Ebinesan AD, Charalambous CP (2013). Metal allergy screening prior to joint arthroplasty and its influence on implant choice: a delphi consensus study amongst orthopaedic arthroplasty surgeons. Knee Surg Relat Res.

[26] Reich J, Hovy L, Lindenmaier H-L (2010). Präklinische Ergebnisse beschichteter Knieimplantate für Allergiker. Orthopäde.

[27] Reiner T, Sorbi R, Müller M (2020). Blood metal ion release after primary total knee arthroplasty: a prospective study. Orthop Surg.

[28] Schafer T, Bohler E, Ruhdorfer S (2001). Epidemiology of contact allergy in adults. Allergy.

[29] Thienpont E, Berger Y (2013). No allergic reaction after TKA in a chrome-cobalt-nickel-sensitive patient: case report and review of the literature. Knee Surg Sports Traumatol Arthrosc.

[30] Thomas P, Schuh A, Ring J (2008). Orthopädisch-chirurgische Implantate und Allergien. Orthopäde.

[31] Tsukamoto R, Chen S, Asano T (2006). Improved wear performance with crosslinked UHMWPE and zirconia implants in knee simulation. Acta Orthop.

[32] Ungethüm M, Winkler-Gniewek W (1984). Metallische Werkstoffe in der Orthopädie und Unfallchirurgie.

[33] van der Veen HC, Reininga IH, Zijlstra WP (2020). Pseudotumours, cobalt and clinical outcome in small head metal-on-metal versus conventional metal-on-polyethylene total hip arthroplasty. Hip Int.

[34] van Hove RP, Brohet RM, van Royen BJ (2015). No clinical benefit of titanium nitride coating in cementless mobile-bearing total knee arthroplasty. Knee Surg Sports Traumatol Arthrosc.

[35] van Hove RP, Sierevelt IN, van Royen BJ (2015). Titanium-nitride coating of orthopaedic implants: a review of the literature. Biomed Res Int.

[36] Vendittoli P-A, Mottard S, Roy A (2007). Chromium and cobalt ion release following the Durom high carbon content, forged metal-on-metal surface replacement of the hip. J Bone Joint Surg Br.

[37] Vertullo CJ, Lewis PL, Graves S (2017). Twelve-year outcomes of an oxinium total knee replacement compared with the same cobalt-chromium design: an analysis of 17,577 prostheses from the Australian orthopaedic association national joint replacement registry. J Bone Joint Surg Am.

[38] Zahiri CA, Schmalzried TP, Szuszczewicz ES (1998). Assessing activity in joint replacement patients. J Arthroplasty.

[39] Zamzow H (2008). Implantatallergien in der Knieendoprothetik aus Sicht eines Unfallchirurgen beim Medizinischen Dienst der Krankenversicherung (MDK). Orthopäde.

